# A qualitative study on the experience of stigma for Chinese parents of children with autism spectrum disorder

**DOI:** 10.1038/s41598-022-23978-0

**Published:** 2022-11-15

**Authors:** Catalina Sau Man Ng, Sally Sui Ling Ng

**Affiliations:** Department of Early Childhood Education, Education University of Hong Kong, 10 Lo Ping Road, Tai Po, New Territories Hong Kong SAR, China

**Keywords:** Psychology, Human behaviour

## Abstract

Experiencing stigma related to having a child with autism spectrum disorder (ASD) can be difficult and is detrimental to parent well-being. Since the research on stigmatized experiences among parents of children with ASD in non-Western communities is limited, this qualitative study examined the experiences, reactions and impacts of stigma on parents of children with ASD in Hong Kong. In-depth interviews were conducted with 54 Chinese parents/caregivers of children with ASD aged between 35 and 73 years old. Data were analyzed using an inductive approach. The participants reported stigma which stemmed from negative labelling of their children by schools and healthcare professionals, bullying by peers, stereotypes of ASD and stigma linked to autistic children’s behavior in the community. The reactions of participants towards stigmatization were classified into internalizing reactions including apologizing, ignoring and concealing ASD and externalizing reactions such as fighting back. The participants also reported impacts of stigma on both personal and emotional levels. The results point to the urgent need for the government to allocate resources and make concerted efforts to reduce stigma by educating the community to foster more positive attitudes towards individuals with ASD and offer support and counselling services to parents.

## Introduction

Autism spectrum disorder (ASD) is a neurological developmental disability that is characterized by restricted, repetitive patterns of behavior, interests, or activities as well as impaired social communication and interaction^1,2^. Since it is a spectrum disorder, affected individuals may have a wide range of symptoms and levels of disability in functioning^3^.

Stigma associated with ASD is widespread^4–6^. Goffman defined stigma as “a phenomenon whereby an individual with an attribute which is deeply discredited by his/her society is rejected because of the attribute. Stigma is a process by which the reaction of others spoils normal identity.”^7^ (p.3). The processes of stigma involve “labelling, stereotyping, exclusion, loss of status, and discrimination”^8^ (p.811). Since individuals with ASD often show an absence of physical markers as a sign of disability but exhibit some behavioral and antisocial behaviors such as tantrums, biting themselves, mouthing objects, and aggression^9,10^, both their parents are blamed for poor parenting which results in displaying inappropriate behavior^11^. Therefore, parents often experience “courtesy stigma”^7,12^ which is “a product of their larger biographical relationship with their child and their known about identity as the parent of a child with disability”^4^ (p.737). Parents of children with ASD are likely to experience enacted stigma which refers to episodes of discrimination or social exclusion against people with a stigmatized condition^13,14^. Parents, particularly mothers, who are the primary caregivers may hold the belief that their child might encounter negative treatment from others if their stigmatized condition is exposed (felt stigma^13,15^). They may internalize public’s negative stereotypes or attitudes as affiliate stigma^9,16^ or internalized stigma^17^. With the increased exposure to negative experiences and feelings of shame or the fear of rejection^9^, the level of psychological distress of associates of stigmatized people increases^16,18^.

A review of the research literature shows that parents of children with ASD reported experiencing different forms of social devaluation and negative treatment from other people at some point in their lives^13^. McCabe reported that most participants experienced discrimination such as despising comments and tease in public places when they were out with their children due to the children’s inappropriate behavior^19^. They felt embarrassed so some parents stopped going out with their children to avoid the uncomfortable interactions that they encountered when their children did not follow social rules^20^. Similar results were reported in a study by Yassibas and Colak as half of the participants said that they did not utilize public transportation to protect themselves from the labelling attitudes of the community but they said this restricted their social life and isolated them from others^21^.

Stigmatizing experiences also can be found in school communities due to a lack of knowledge regarding autism^22^. In Hong Kong, integrated education was adopted by the Education Bureau. All students are included in ordinary schools regardless of any special needs except for those with more severe or multiple disabilities who require intensive care and support. Hence, children with ASD without intellectual disabilities attend mainstream schools^23^. To effectively teach children with ASD, teachers need to be equipped with appropriate knowledge and attitudes. However, although special education training has been offered, only 10% of primary and secondary teachers in Hong Kong have enrolled in those training programmes in the past 10 years^24^. It is therefore not surprising that teachers in Hong Kong were found to be insufficiently trained and inadequately skilled to teach children with special education needs (SEN)^25,26^.

Emerging empirical evidence shows that the degree to which ASD is stigmatized varies greatly across cultural contexts. Individuals in collectivist cultures tend to hold more stigmatizing attitudes towards people who deviate from the norm such as those affected by ASD and mental illnesses^27^. They tend to have less tolerance towards diversity than in individualistic cultures^27,28^. For example, college students from Lebanon and Japan where collectivist culture is dominant reported higher levels of stigma towards ASD than those from the United States, a country with an individualistic culture^29,30^. Abdullah and Brown found that the Asian and African American cultural groups had stronger stigmatizing beliefs than other American cultural groups^31^. Studies have found that with the strong influence of Confucianism, Buddhism, and Taoism, Chinese parents of children with ASD tend to be more susceptible to self-stigma^32,33^ because they focus more on their social identity and “face concern”^16,34,35^.

With the increasing prevalence of ASD globally^36^, there are reasons to assume that the number of stigmatized individuals and their associates is substantial. It is also important to understand the real-life stigmatized experiences encountered by parents of children with ASD because not only does stigmatization cause mental health problems such as anxiety and depression in parents of children with ASD^37^, but it may also have spillover effects on their family members such as their spouses^4,38^. For example, Zhou and Yi found that the emotional states of parents of children with ASD have some influence on children’s emotions and behaviors^39^. Therefore, understanding the stigmatized experiences encountered by parents of children with ASD can provide us with insights on how to reduce stigma in society.

Fabrega commented that stigma seems to be less evident in Asian and African countries^40^. However, Corrigan and Watson argued that it is likely due to a lack of studies in these countries^41^. Our study aimed to fill the knowledge gap by investigating stigma in Hong Kong, which has a strong collectivist culture. In addition, to date, most studies utilized Western samples^42^ and the results are not applicable to Asian samples due to cultural differences. To our knowledge, the present study is among the first to investigate stigma in Chinese parents of children with ASD in Hong Kong using the qualitative method as most existing local studies used the quantitative method^16,43^. Our qualitative data will contribute to increasing the currently limited knowledge of the experiences of stigmatization in Chinese parents of children with ASD and provide a direction for reducing stigmatization.

## Methods

### Study design

The present study was part of a larger study which examined the predictors of difficulties in reading comprehension in children with ASD. We aimed to explore the stigmatizing experiences of parents having a child with ASD. A qualitative study design was employed because it provided complex descriptions of how individuals experienced a phenomenon, reacted to it, as well as what impact stigma had on them.

### Participants

The participants were parents whose children were diagnosed with ASD by healthcare professionals. The inclusion criteria for participation were as follows: (1) aged 18 or above, (2) ethnically Chinese, (3) Cantonese speaking, (4) having a child aged between 6 and 12 years old who was diagnosed with ASD by healthcare professionals. The exclusion criterion was: having a child with special education needs other than ASD such as attention deficit hyperactivity disorder (ADHD) or learning difficulties or visual or hearing impairment.

We successfully recruited 61 Chinese parents/caregivers of children with special education needs to join our study. Among them, the parents/caregivers of seven children were excluded because they were diagnosed with ADHD or dyslexia, not ASD. A total of 38 females (70.37%) and 16 males (29.63%) participated in the study. They ranged in age from 35 to 73 years, with an average age of 45.04 years (SD = 7.30) and a median age of 44.0 years. The detailed demographic characteristics of the 54 parent participants from 43 families are summarized in Table 1. The mean age of children (boys: 38; girls: 5) with ASD participating in our study was 9.79 years (SD = 1.34), ranging between 7 and 12 years and a median age of 10.0 years.

**Table 1:** Demographic characteristics of the participants (N = 54).

Age	N	(%)
Mean (± SD)	45.04 (± 7.30)	
Median	44.0	
Missing	6	(11.11)
**Gender**		
Female	38	(70.37)
Male	16	(29.63)
**Relationship to child with ASD**		
Mother	37	(68.52)
Father	16	(29.63)
Grandmother	1	(1.85)
**Marital status**		
Married	51	(94.44)
Divorced	2	(3.70)
Widowed	1	(1.85)
**Educational level**		
Primary or below	1	(1.85)
Secondary	26	(48.15)
Diploma	5	(9.26)
Bachelor’s degree	10	(18.52)
Master’s degree or above	8	(14.81)
Missing	4	(7.41)
**Occupation**		
Housewife	15	(27.78)
Professional	11	(20.37)
Management	7	(12.96)
Customer Service	6	(11.11)
Clerk	4	(7.41)
Assistant professional	4	(7.41)
Technician	2	(3.70)
Retired	1	(1.85)
Missing	4	(7.41)
**Child with autism (N = 43)**		
**Gender**		
Son	38	(88.37)
Daughter	5	(11.63)
**Age**		
Mean (± SD)	9.79 (± 1.34)	
Median	10.0	
Missing	1	(2.33)

### Data collection and management

Parents/caregivers were recruited through a poster on social media for the recruitment of children under the age of 12 to do a reading comprehension test. On the poster, we also recruited eligible parents to attend an in-depth interview while their children were taking the test on reading comprehension. However, if parents refused to join the interview, this would not affect the chance of their child participating in the reading comprehension test. We promised to send a detailed reading report including their children’s weak areas to parents after the data analysis was completed. The interviews took place between March 2018 and January 2019.

On the test date, while their child was having the test, the eligible consenting parents joined the interviews. If both parents accompanied the child and agreed to join the interview, they were given the choice of being interviewed together or individually. All parents preferred to have joint interviews. Prior to the interviews, all participating parents were told about the objectives of the study, they were informed of the voluntary nature of their participation in the study, their right to withdraw from the study at any time and the confidentiality of the data. Written informed consent was obtained from all participants. The methods used in this study were carried out in accordance with relevant guidelines and regulations. We sought ethical approval and followed all ethical procedures for research set by the Human Research Ethics Committee (HREC) of the Education University of Hong Kong.

The interviews were conducted by a moderator who was either CSMN or a trained project assistant with a bachelor’s degree in psychology studying a master’s degree in speech therapy. Both moderators were female and they shared all the interviews equally. A trained assistant moderator took field notes during the whole interview process. The interviews lasted between 45 and 90 min and were carried out in an interview room at the university campus. All the participants were given a letter (e.g., A, B, C, etc.) during the interview to protect their identity. The data collection was directed by a semi-structured interview guideline (see Appendix 1). The interview questions included the following: (1) Have you experienced any stigma because of your child with ASD? Could you share your experience with us? (2) What were the reactions and impacts of stigmatized experiences on you?.

This qualitative study involved 43 in-depth interviews with 54 participants. Among 43 interviews, 11 were joint interviews (i.e., ten interviews were conducted with both parents while one interview was conducted with two aunts of the child with ASD). During the joint interviews, for each question, the moderator checked each parent’s perspective on the issue(s) to allow them to have the opportunity to express their views individually^44^. Therefore, both parents’ views were included in the data analysis. All interviews were audio-recorded with written consent from all the participants. Data collection was discontinued when data saturation was reached.

### Data credibility

We adopted several strategies to enhance the credibility of the data collection. Prior to the data collection, both moderators reviewed and discussed interview techniques such as how to ask the questions and how to use probes to elicit the relevant information^45^. The use of different moderators can minimize the bias associated with having one moderator conducting all the interviews^46,47^. During the data collection, both moderators had regular meetings to reflect on the interviews which enabled the interviews to gain more depth as the data collection was progressing^48^.

### Data analysis and research rigor

All audio recordings were saved in an encrypted file. The content of the interviews was fully transcribed verbatim in Cantonese to capture the nuances of expression specific to this Chinese dialect, which is the native dialect of the moderator, assistant moderator, and all the participants. All identifiable personal information was removed from the text to ensure confidentiality. The trained assistant moderator performed quality checks by comparing the recordings to the transcripts. Only the quotes cited in the present manuscript were translated into English.

We adopted a five-step inductive approach which links the data analysis to the specific research questions^49^. We first conducted a careful, initial reading of the transcripts, followed by the identification of specific text segments related to research questions, labelling the segments of the text to create categories, the elimination of the redundancy among the categories, and lastly, the creation of a model which incorporates the most important categories^50^.

To confirm the reliability of the coding process, both researchers (CSMN and SSLN) read all the interview transcripts independently to familiarize themselves with the data. Several meetings were held to discuss and code the interview transcripts based on the semi-structured interview guideline as the framework. They compared their initial coding of two identical transcripts and refined the analysis framework. Then SSLN coded all the transcripts based on the refined analysis framework. To ensure the reliability of the coding, intercoder reliability (ICR) was performed. Prior to performing the ICR, each transcript of each interview was given a number (e.g., 1, 2 etc.) and the transcripts were randomly selected for inter-rater reliability based on the numeric values assigned to the respective transcripts. As a result, 20 (46.51%) randomly selected narratives from transcripts were coded by CSMN. Her coding was compared with the coding by SSLN. The intercoder agreement percentage on the codes was 90%. Regular meetings were held to discuss any doubtful coding.

## Results

**Figure 1:**
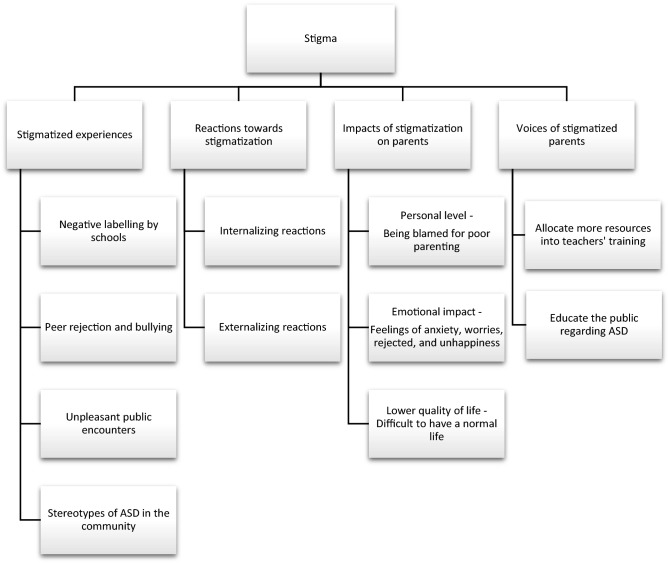
Themes and subthemes of individual interviews.

Among 54 participants, 40 participants (74.07%) expressed that they encountered some experience of stigma at some point in their lives, while 14 participants (25.93%) reported that they never had such experience because their children behaved well in public places. Therefore, the data of the 14 participants were not included in the themes and sub-themes which are summarized in Fig. 1 and Table 2. The results consisted of four main themes: (1) stigmatized experiences, (2) reactions towards stigmatization, (3) impacts of stigmatization on parents and (4) voices of stigmatized parents.

**Table 2:** Theme-subtheme endorsement by gender and educational levels of parents/caregivers (N = 40).

	Female (N = 31)							Male (N = 9)					Total (N = 40)	
	Secondary	Diploma	Bachelor’s degree	Master’s degree or above	Education level not reported	Sub- total	%^^^^	Secondary	Bachelor’s degree	Master’s degree or above	Sub-total	%^^^^	Total	%^^^^
**Theme 1: Stigmatized experiences**														
(1) Negative labelling by schools	1		2			**3**	***7.5***	1	1	1	3	7.5	**6**	***15.0***
(2) Peer rejection and bullying	4	2	1		2	**9**	***22.5***			1	1	2.5	**10**	***25.0***
(3) Unpleasant public encounters	8		3	2	1	**14**	***35.0***	1	1		2	5.0	**16**	***40.0***
(4) Stereotypes of ASD in the community	2	1			1	**4**	***10.0***	1	1		2	5.0	**6**	***15.0***
**Theme 2: Reactions towards Stigmatization**														
(1) Internalizing reactions	3		4	1		**8**	***20.0***				0	0.0	**8**	***20.0***
(2) Externalizing reactions						**0**	***0.0***		1		1	2.5	**1**	***2.5***
**Theme 3: Impacts of stigmatization on parents**														
(1) Personal level–Being blamed for poor parenting	7	1	1		2	**11**	***27.5***	1			1	2.5	**12**	***30.0***
(2) Emotional impact–Feelings of anxiety, worries, rejection and unhappiness	4	1	2	1	1	**9**	***22.5***	1			1	2.5	**10**	***25.0***
(3) Lower quality of life–Difficult to have a normal life	4		2			**6**	***15.0***				0	0.0	**6**	***15.0***
**Theme 4: Voices of stigmatized parents**														
(1) Allocate more resources into teachers’ training	4	1	1			**6**	***15.0***		2		2	5.0	**8**	***20.0***
(2) Educate the public regarding ASD			1			**1**	***2.5***	1	1	1	3	7.5	**4**	***10.0***

^^ = The calculation of the percentage is based on the total number of participants (N = 40).

### Theme 1: stigmatized experiences

#### Negative labelling by schools

Six participants reported that they experienced stigma at school as their children with ASD were perceived and labelled as having low learning abilities. Some parents expressed that their children with ASD were even asked to leave their school because most teachers were not knowledgeable enough to teach children with ASD. In addition, the schools reiterated that teachers teaching children with special needs should be kind and patient, which are not qualities shared by all teachers. Some participants were told to provide additional support such as hiring a shadow teacher to assist their children’s learning if they continued to study at schools.

“The school has started… to find some reasons to reject us. There were a few signs at the end of the second semester. My wife is the main contact. In fact,... I felt that the school wanted us to withdraw from school (because my son has ASD). The school said something like this: "If you want to attend class, we had many similar cases... Maybe you can find a shadow teacher to sit next to him. But you need to understand that the cost is high." The school told us many things like this.” (Participant 1, Father, 43, Master’s degree or above; Son, 10, ASD).

“I must explain to the school clearly. My son has autism... He is now studying in a school for people with mild intellectual disability. “Do you mind teaching him?” Nine out of ten schools said no. It’s very difficult to find a teacher who is kind and patient. They may charge us more money for teaching him. Difficult!” (Participant 45, Father, 54, Bachelor’s degree; Son, 12, ASD).

A participating parent shared that her child was placed on the waiting list of the school’s sports team for several years but still was not selected. Her child with ASD felt frustrated and lost motivation to join the sports team.

“My son applied for joining the volleyball team, but his teacher did not select him. The teacher said that he could join the school’s sports team in the first year. However, many years have passed, and he still could not join the team. The teacher did not directly reject him but placed him on the waiting list. My son is so disappointed that he does not want to join the team anymore.” (Participant 51, Mother, 39, Bachelor’s degree; Son, 10, ASD).

One of the participating parents expressed that stigma not only comes from the schools, but also from professional people like the medical personnel when they dealt with their child’s issues.

One participant narrated what the psychiatrist said which showed discrimination.

“My child was suddenly excluded from participating in a freestyle relay swimming competition, but we did not receive any prior notice about his exclusion from joining the competition. A psychiatrist blamed me that I should not entangle with the school because of the issue. He said, “The school has already given up this group of students. What was the point of contacting the school for this?”.” (Participant 52, Mother, 43, Bachelor’s degree; Son, 12, ASD).

#### Peer rejection and bullying

Some children with ASD have problems in making eye contact with others, which interferes with their everyday social interactions. They cannot interact well with other children and other children may reject them so they have more difficulties in making friends.

“One of the biggest problems for children with ASD is that other children do not play with him. It’s very clear and his school also knows that he has no friends. He walks and looks around during recess.” (Participant 1, Father, 43, Master’s degree or above; Son, 10, ASD).

“During playtime, the classmates said, “Don’t come, don’t come, you cannot play well.” Therefore, no one likes playing with him. In fact, my son is eager to play with them.” (Participant 47, Mother, 39, Secondary; Son, ASD).

In our study, ten participants reported that their children were bullied and rejected by classmates at schools. A mother expressed that her son was rejected because he showed some odd behaviors, did not speak and look at others.

“There are some levels of rejection to my son. They find him weird as he has many odd behaviors, no sounds and eye contacts.” (Participant 33, Mother, 44, Secondary; Son, 8, ASD).

“Other classmates usually keep a distance from her because they are afraid of being teased by others.” (Participant 34, Mother, 47, Bachelor’s degree; Daughter, 11, ASD).

A mother described in detail how her son was bullied by other students. She described that her son’s classmates dragged him to the tuck shop and told him to pay for the snacks. Sometimes his classmates even drew pictures on his shirt (uniform) and school bag or stole his stationery.

“The school allows students to use Octopus card to buy food from the tuck shop. A classmate asked my son to treat him and he did. After this, they didn’t ask my son to treat them. What they did was that they dragged him to the tuck shop and waited for him to pay. They even took his Octopus card from his school bag to buy snacks… Suddenly, a child at the back had nothing to do, so he just took a pen to draw pictures on his shirt.” (Participant 16, Mother, 40, Secondary; Son, 8, ASD).

“At the beginning of the school year, I found he lost many personal belongings. Besides, he did not tell me immediately after being bullied by classmates at school.” (Participant 19, Mother, 42, Diploma; Son, 9, ASD).

Due to the inability of children with ASD to express themselves in an organized way, most of them failed to describe the bullying situation in detail. The bullying may have lasted for a long time because no teachers or no parents were able to find out until the children with ASD voiced it out. Worse still, they did not report the bullying situation immediately.

“When my son was in grade 3, collective bullying happened to him. At the beginning, I found he lost a lot of personal stuff. His things were torn into pieces, and sometimes were glued. Due to his inability in self-care, I blamed him several times and I was not aware of bullying. Until the situation got more serious which drew my attention… For those ASD children being bullied at school, they do not report to parents immediately. They are upset and do not know how to speak. It may take them a month to organize the details of what happened about the bullying.” (Participant 19, Mother, 42, Diploma; Son, 9, ASD).

“When my son was in grades 1 and 2, he was bullied by two classmates with special needs. They dragged my son outside the classroom and beat him several times. Since my son has autism and his personality is fairly gentle, I did not know that he was often beaten as he did not reveal it to me…” (Participant 44, Mother, 37 Secondary; Son, 9, ASD).

“My son did not tell his teachers that he was bullied. I asked him why and he explained to me that his classmates told him not to tell the teachers.” (Participant 58, Mother, Age not reported, Daughter, 9, ASD).

#### Unpleasant public encounters

Sixteen participants revealed some unpleasant public encounters like being stared at or being gossiped about when they went out with their children with ASD in public places such as parks, markets, or restaurants because their children showed some socially unacceptable behaviors such as beating a table, making loud noises in restaurants or lying on the ground in a wet market during a temper tantrum.

“For example, when we go to the park, she often displays some odd behaviors that make people stare at us. Therefore, when there are many people, we don’t dare or don’t want to play there.” (Participant 34, Mother, 47, Bachelor’s degree; Daughter, 11, ASD).

“Here was my experience in a wet market where I went shopping with my son. He showed his temper tantrums by lying on the ground near a fish stall. At that time, many people were in the market. Of course, they saw and were talking about us… about my son’s behavior. I couldn’t stop them talking as I needed to handle my son first.” (Participant 7, Mother, 44, Secondary; Son, 8, ASD).

“[I] Went out with him… other people often stare at us… that is, people think that you made a lot of noise while you were eating. Why did the child get the bowls in a bouncing and jumping way? Why did he beat the table loudly?” (Participant 31, Mother, 37, Secondary; Son, 7, ASD).

#### Stereotypes of ASD in the community

Six participants expressed that some people still have some stereotypical perspectives towards individuals with ASD such as considering them as “stupid”, “mentally ill” or “silly”. The public does not accept individuals with ASD so it is difficult for them to integrate into society including for something as simple as going out. There is evidently strong social disapproval in the community.

“Although people have a better knowledge of ASD and the media also introduce it, some people always think that they are problematic. People always think that individuals with ASD are stupid, mentally ill, and incurable. There are many different misunderstandings. Even some people would think, “ASD, so what? Is it only people with ASD allowed to do this?” I have heard of many remarks like this.” (Participant 21, Mother, Age not reported, Secondary; Son, 12, ASD).

“Many people in society still don’t accept them. For example, when my son was a child, he often messed with other people’s things. I apologized and said that my child was too young to be aware of that. I remembered once someone yelled at me, “He is silly. You shouldn’t take him out!”.” (Participant 6, Mother, 47, Secondary; Son, 10, ASD).

“The society cannot accept children with special needs.” (Participant 20, Mother, 46, Diploma; Son, 10, ASD).

### Theme 2: reactions towards stigmatization

#### Internalizing reactions

In response to unpleasant public encounters, the participants often gave an immediate apology to those who were offended to refrain from escalating negative feelings. Two participating parents expressed that they would teach their children to apologize when they were in such a situation.

“When people scolded me, I apologized immediately.” (Participant 6, Mother, 47, Secondary; Son, 10, ASD).

“When my son bumped into other people, I would teach him to apologize and tell him not to disturb others again. I think that since I gave birth to a son like this, I need to teach him. If he does not know, he needs to learn.” (Participant 12, Mother, 60, Bachelor’s degree; Son, 11, ASD).

Two parents shared that they could tolerate some negative comments.

“As he is my son, I can accept his deficiencies. When he was young, many people said that he was so naughty and behaved uncontrollably. I could stand such negative comments on my son’s problem behavior.” (Participant 21, Mother, Age not reported, Secondary; Son, 12, ASD).

In addition, a parent expressed that when she was scolded or faced negative criticism from other people, she would pretend not to hear the harsh criticism and did not show any responses by remaining silent so those people would stop criticizing.

“Yes, I have been scolded by other people. It’s very uncomfortable to hear that. I pretended not to hear it, [laughing] and see it. Really pretending not to hear anything is good. In fact, they just say a few sentences and then, will stop. You shouldn’t respond to them unless you want to argue with them which will take a long time. If you don’t say anything, they will not continue to talk too long. They only talk about the same thing repeatedly. If you don’t speak out, they will stop.” (Participant 34, Mother, 47, Bachelor’s degree; Daughter, 11, ASD).

Another reaction to stigmatization is to conceal the truth to protect children from being labelled. Labelling is a kind of stigmatization. Three participants expressed that they would protect their children from labelling by concealing the diagnosis of ASD of their children from schools. The labelling of children with ASD is a major concern for those parents. They were worried about the effects that labelling could have on their children. The consequences may be detrimental to the well-being of their children. Labelling creates negative stereotypes in relation to the disabilities of individuals with ASD such as their lower intelligence quotient (IQ) and learning disabilities. To protect their children from being labelled, those three participating parents indicated that they preferred not to disclose the ASD diagnosis of their children to anyone including schools and relatives if the ASD symptoms of their children were not obvious.

“The ASD symptoms of my child are not so obvious that they can alert the teachers. Besides, I don’t think that my child needs any support. Therefore, I did not tell the class teacher about the ASD diagnosis of my child to avoid being labelled by the school.” (Participant 43, Mother, 48, Secondary; Son, 11, ASD).

#### Externalizing reactions

Not all parent participants had internalizing reactions. A parent expressed that he reacted differently by fighting back (i.e., scolding) the person who showed discrimination to his son.

“Sometimes when I meet people who show discrimination to us, I scold them.” (Participant 45, Father, 54, Bachelor’s degree; Son, 12, ASD).

### Theme 3: impacts of stigmatization on parents

#### Personal level—Being blamed for poor parenting

Twelve participants of children with ASD reported that they had been accused of poor parenting which caused the misbehavior of their children. They also received some “unsolicited” advice about their parenting, including “don’t take care of him anymore”.

“His odd behavior makes people misunderstand that it is due to poor parenting. Parents don’t teach.” (Participant 6, Mother, 47, Secondary; Son, 10, ASD).

“The teacher keeps on telling me, "It's because of you... that makes your child become worse." He told me that my child’s self-care skills are poor because of me. "You take care of him all the time so he doesn’t know how to take care of himself. If you don’t take care of him, he will learn and master the skills. Don’t take care of him anymore..." At that time, I thought that I should leave him alone because the teacher kept on telling me, telling me every day, and keeps on saying that it’s because of me. "You harm your son." I have heard it many times.” (Participant 20, Mother, 46, Diploma; Son, 10, ASD).

“At school, my son often rocks a chair, never sits still, and doesn’t care about other people. When the teacher met me, she said that I let him do whatever he wanted.” (Participant 48, Mother, Age and education not reported; Son, 11, ASD).

#### Emotional impact—Feelings of anxiety, worries, rejection and unhappiness

Ten participants shared that they experienced anxiety and worries when they went out with their children with ASD because of their behavior. When they heard other people’s comments, they felt unhappy and were sad about the situation. They also felt rejected and excluded by society. A mother expressed that she was exceptionally worried that her son’s inappropriate behavior may end up being spread on social media as the sharing of videos on social media such as Facebook, YouTube and Instagram is very popular nowadays.

“[I] Keep on reminding him. Sometimes I feel anxious when I go out. I’m so worried that something might happen, or he may show some bad behavior.” (Participant 5, Father, 51, Secondary, Son, 11, ASD).

“Actually, the best way is that people around them [individuals with ASD] ignore and do not react to the anti-social behavior of my child and pretend that nothing is happening in any embarrassing situation. I hope my son’s odd behavior will not be put on social media. Mothers of children with ASD have a lot of pressure.” (Participant 12, Mother, 60, Bachelor’s degree; Son, 11, ASD).

“Parents of children with autism are very uncomfortable about people’s comments. They hear their comments… and feel very unhappy.” (Participant 21, Mother, Age not reported, Secondary; Son, 12, ASD).

#### Lower quality of life—Difficult to have a normal life

Since children with ASD often show some socially unacceptable behavior and can sometimes even be out of control, six participants said that they avoided taking their children out to play with friends as well as to dinner. They preferred to stay at home to avoid stigmatized experiences but as a result, they cannot have a normal life and feel a sense of loneliness.

*“When we dined out, we tried to eat faster or ordered take-away so as to avoid other people’s complaints about my child’s odd behavior in public areas.”* (Participant 31, Mother, 37, Secondary; Son, 7, ASD).

“Don’t like to take him out.” (Participant 6, Mother, 47, Secondary; Son, 10, ASD).

“I need to feel that I can control the situation before we go out. This makes me feel I am socially isolated. I don’t want to take him out. Even if I took him out, he would not play with other children, just walking around.” (Participant 38, Mother, Age not reported, Secondary; Son, 9, ASD).

“I think the hardest thing is to live a normal life. I want to go out and enjoy life normally.” (Participant 34, Mother, 47, Bachelor’s degree; Daughter, 11, ASD).

In addition, when they went out with their children with ASD, they felt under pressure and nervous. They even felt that everyone was looking at them. One participant further shared that she preferred to talk and stay with people whose children have ASD as she felt they were more understanding of the problems of children with ASD. She did not feel comfortable staying with people whose children had a normal development because they did not understand her.

“In fact, as soon as you take him out, you will feel that everyone is looking at you. Then there is some pressure.” (Participant 12, Mother, 60, Bachelor’s degree; Son, 11, ASD).

“I take my son out only for treatment and training. Honestly, I don’t want to take him out for shopping as I am very anxious about his odd behavior... I will take him out to meet friends who are also parents of children with ASD. I feel easy and comfortable because they accept my child. I am not willing to meet parents who do not like me. Actually, I am very lonely. I hardly talk with parents whose children don’t have ASD, as they might not understand my child’s problems.” (Participant 47, Mother, 39, Secondary; Son, Age not reported, ASD).

### Theme 4: voices of stigmatized parents

#### Allocate more resources into teachers’ training

Eight participants have stressed that most teachers lacked knowledge of ASD and time to handle children with ASD. Increasing teacher professional training on special education needs is urgently needed, especially enhancing knowledge to eliminate misconceptions and increase their awareness of the needs of individuals with ASD.

“Teachers of the mainstream kindergartens cannot handle these students. They don’t have time to teach them and manage them well.” (Participant 32, Father, 43, Bachelor; Son, 8, ASD).

“Teachers’ knowledge of how to educate children with ASD is insufficient. We interacted with some teachers who did not understand the needs of children with ASD. Teachers also discriminate students with ASD. I heard that one of the teachers said, ‘ASD is a kind of learning deficiency.’” (Participant 49, Mother, 35, Secondary; Son, 9, ASD).

“[Students with ASD] sometimes make their teachers angry unintentionally. If teachers in mainstream schools are not experienced and skillful in managing the situation, they may not get along with each other well.” (Participant 56, Mother, 43, Secondary; Son, 11, ASD).

#### Educate the public regarding ASD

Four participants have called for public education on ASD as they believe that the public is still ignorant about ASD and that stigmatization could be reduced by enhancing the public’s knowledge about children with ASD.

“… There is a lot of discrimination against individuals with ASD. Among people who show discrimination, some people are not highly educated so they may have little knowledge about autism… I think inclusive education is good; however, the Government did not allocate sufficient resources into it.” (Participant 45, Father, 54, Bachelor’s degree; Son, 12, ASD).

“Educating the public is deemed necessary. I hope the public can accept children with ASD and have better knowledge about them. I wish the public did not discriminate against them anymore.” (Participant 12, Mother, 60, Bachelor’s degree; Son, 11, ASD).

## Discussion

A qualitative approach was employed to explore real-life experiences, reactions and impacts of stigmatization among 54 Chinese parents who have a child with ASD, thus allowing for a better understanding of stigmatizing experiences and how stigma affects them in a non-Western context which is missing in the existing research literature.

There were 54 participants joining the interviews with a total of 43 interviews, out of which 11 were joint interviews. As both parents were present in joint interviews, the mother often turned to the father to check the accuracy of her responses, which in turn, enhances data accuracy. In addition, the perspectives of fathers could also be elicited and compared with those of mothers to provide richer data^51,52^. Therefore, the paternal presence has improved data quality and enriched data. That being said, researchers should be aware of the order of response in the joint interviews which may result in an unequal voice of one parent. In our study, mothers often responded first so some responses may not have been reiterated again by fathers which may result in low theme endorsement.

Forty participants in our study expressed that they experienced stigmatization. Their cited real-life situations showed that they often experienced enacted stigma because of subtle forms of social devaluation such as being stared at, being treated unkindly, or being avoided^15^. However, 14 participants revealed that they did not experience stigma. One of the plausible explanations for the difference in the degree of exposure of stigmatized experiences is the level of severity and visibility of children’s ASD symptoms. The 14 participants reported that their children did not show socially inappropriate behaviors in public situations due to mild symptoms of ASD. These results corroborated the results of some Western studies^4,10^ which found that stigma is related to the severity and visibility of special education needs. Therefore, participants with children who exhibit more symptoms of ASD may have a higher chance of being stigmatized. Since ASD is considered as a general diagnosis in the present study and the level of the severity of the ASD symptoms has not been considered here, future studies are needed to ascertain the relationship between the degree of severity of ASD and stigmatized experiences. Another possible reason is that some parents may have experienced stigma but they did not want to report that they felt stigmatized (reporting bias) so they may be more likely to report that their child did not show socially inappropriate behaviors in public situations.

The results of our study revealed that participating parents experienced courtesy stigma because of their close relationship with the stigmatized individuals^53^ which made them the targets of stereotypical opinions and discriminatory behaviors^54^. Stigmatized experiences including negative labelling, stereotyping, isolation, and discrimination encountered by the participants were not only brought about by the public who may be less knowledgeable about individuals with special needs, but surprisingly, were also caused by highly educated people such as professionals in the educational and medical fields. Our qualitative results indicated that schools labelled children with ASD as “learning disabled”^55,56^. These negative stereotypes linked to labelling differences are a form of stigma^57^. The stereotypical view is that people regard individuals with special education needs as generally incapable. However, Maenner and colleagues found that about 70% of individuals with ASD show average to above average intellectual functioning while only 30% meet the criteria for intellectual disability^58^. Hetzroni et al. reported that some individuals with ASD can be creative^59^. Clearly, the stereotypical view about ASD shows that educating the public is urgently needed.

Our qualitative results revealed the range of stigmatized experiences of children with ASD who were either excluded from participating in a swimming competition, placed on the waiting list of sports teams for years or were asked to leave their school. Negative perceptions of children with special needs can be damaging because such perceptions influence the perceptions of others towards individuals with special needs^60^ which could impact the way individuals with ASD are treated at school and limit the opportunities available to them.

As in other studies^61,62^, the experiences of discrimination such as bullying at school were frequently reported by participating parents. The situation can be partly explained by the contrast between the absence of physical markers in most children with ASD and their odd behavior^4,63^. Zablotsky et al. found that over 60% of children with ASD had experienced victimization some time in their lives^64^. Evidently, younger children like primary school children did not know what ASD is or had no relevant knowledge of it^65^, which caused them to develop negative attitudes towards children with ASD, which in turn has a negative impact on the inclusion of children with ASD at school^66^. More concerted efforts are needed from schools to create a safe environment where all children can thrive, socially and academically.

Many participants expressed that the types of inappropriate behaviors exhibited by children with ASD such as screaming, tantrums or meltdowns and head-banging contribute to societal stigma^10,67^. It is in such public situations that judgements are made about the parents’ ability in child rearing and that the child with ASD displays what is seen as an abnormal behavior, which makes the parents feel embarrassed^68^. As in other studies^63^, we also found that the participants were criticized for their inability to discipline their children or their poor parenting. These experiences made the participants feel judged, humiliated, and socially rejected^27,69^. In Chinese culture, poor parenting (mei jiao yang) is a serious criticism which brings shame and loss of ‘face’ not only to the parents but also to the whole family^70^. To avoid encountering embarrassing situations in which their children may exhibit an abnormal behavior, the participants preferred to stay at home^71^. This strategy, either used on its own or in combination, provides parents of children with ASD with some degree of protection against stigmatized experiences. However, this strategy seems to be a double-edged sword because it disturbs their social life^72^ and as a result, the quality of life (QOL) of parents and children with autism is compromised^9,73^.

Based on our findings, some participants chose to conceal their situation from others. This strategy can only work for children with mild autistic symptoms which people are unaware of. However, not disclosing the truth may prevent them from seeking help from others. Concealing the condition of children with ASD involves a huge effort and planning to keep the truth hidden may result in higher levels of stress and anxiety^74^. Future studies can explore the relationship between concealing behaviors and internalizing symptoms over time.

It is interesting to note that there are some differences in reactions towards stigma: internalizing and externalizing reactions. Evidently one of the concerns of the participants is the potentially negative consequences of their anger expression. They ignore the comments because they are afraid that speaking out may escalate the situation and result in other people uttering more unpleasant comments so they ignored them. Regarding externalizing reactions, since only one participant reported that he would fight back, future studies can further investigate the different reactions towards stigmatized experiences.

Participating parents revealed that some people believe that individuals with ASD are “stupid”, “mentally ill” or “silly”, which reflects the public’s negative perception and stereotypical views concerning ASD as well as a poor knowledge of ASD, in line with the findings in the existing literature^5,9^. Firstly, educating the public via mass media and some educational campaigns to reduce stigma and negative perceptions towards individuals with ASD is urgently needed. Secondly, to help families in particular, more resources providing tangible and intangible support, including emotional and esteem support to parents of children with ASD are needed. Thirdly, the government should allocate more resources into providing professional training to teachers to enhance their knowledge and skills for teaching students with special education needs. Alternatively, universities should equip students with the knowledge and skills of how to teach students with special education needs in their teacher education training.

Apart from experiencing courtesy stigma, participating parents internalized the stigma. They felt sad, anxious and rejected. In addition, parents of children with ASD have very limited social circles as they prefer to socialize with families who also have children with ASD as they show more understanding. The “avoidance by others and the resulting limitations on the abilities of the families to socialize was often felt by the parents to be a considerable loss”^4^ (p.741). Issues related to the internalization of stigma need further investigation as the mental health of parents of children with ASD is of great concern.

### Limitations and future research

The present study has limitations. Firstly, over 70% of the participants were mothers. Mothers may perceive more stigma because they are given the primary responsibility of taking care of the children. Therefore, it is possible that mothers may encounter more negative reactions from outsiders. Secondly, given that the samples were Chinese parents from Hong Kong, which is a metropolitan city, the results may not be generalizable to other populations from other cultures and rural areas. Future studies should examine stigma using samples from non-Western communities and rural areas to ascertain the findings. Thirdly, as the level of severity of the ASD symptoms, which has often been reported as a predictor of the stigma experienced by parents/caregivers has not been considered here, future studies should include the level of severity of children with ASD and explore its relationship with stigma. Finally, apart from retrospective interviews, future studies can also use longitudinal qualitative studies to capture the experience of stigma of parents of children with ASD over time.

## Conclusions

Our study contributes important information about the experiences and reactions of Chinese parents of children with ASD in relation to stigmatization and the impact it has on them. The results point to more concerted efforts and resources that are urgently needed by the government to provide culturally specific interventions, support and counseling for parents/families with children with ASD, organize more campaigns to educate the general public about ASD, provide more professional development training courses for in-service teachers and recruit more social workers to serve in educational settings to assist children with ASD.

## Supplementary Information

Supplementary Information.

## Data Availability

Data are the transcripts of interviews containing personal information. Data are not publicly available due to concerns that the participants’ privacy may be compromised. Anonymized and de-identified data may be requested from the corresponding author.
