# Supplementary material for: A qualitative study on the experience of stigma for Chinese parents of children with autism spectrum disorder
Source: Sci Rep. 2022 Nov 15;12:19550. doi: 10.1038/s41598-022-23978-0 (PMC9666461; doi:10.1038/s41598-022-23978-0)
Supplement: Supplementary file 1 — Supplementary Information. [file 41598_2022_23978_MOESM1_ESM.pdf]

Interview Guideline

(I) ***Experiences of having a child with Autistic Spectrum Disorder (ASD)***

1. (i) When was your child diagnosed with ASD?
   (ii) What were the symptoms exhibited by your child?
2. How did you and your immediate family respond to your child's diagnosis?
3. (i) How has your child's diagnosis changed your
   (a) personal life including career, leisure time etc.;
   (b) family life;
   (c) family routine;
   (d) spousal relationship (e.g., more disagreements/conflicts between you and your spouse)?
   (ii) Have you experienced any stigma due to having a child with ASD? Could you share your experience with us?
   (iii) What were your reactions to stigmatized experiences and what impacts did those experiences had on you as parents of children with ASD?

(II) ***Parental stress***

1. (i) What stressors have you experienced raising a child with ASD?
   (ii) How did you deal with the stress?
   (a) Which methods of dealing with stress were effective? Which ones were ineffective? Could you share your experience with us?
2. (i) On a scale of 0-10 (0=no quality of life; 10=high quality of life), how do you perceive your quality of life with a child having ASD?
   (ii) What prevents you from having a high quality of life? Could you share your experience with us?
3. (i) What difficulties are you facing when taking care of a child with ASD?
   (ii) What kind of parenting have you used with your child with ASD? Why?

(III) ***Supportive measures for ASD parents/families***

1. (i) What kind of information did you need immediately following your child's diagnosis with ASD? Why?
   (ii) On a scale of 0-10 (0=strongly dissatisfied and 10=very satisfied), how satisfied were you in terms of the quantity and quality of the information about ASD which you received after your child's diagnosis from the healthcare professionals and relevant departments in Hong Kong? Why?

2. What kind of support do you need when you take care of your child with ASD?

3. (i) Do you think the support provided to families with autistic children from the relevant departments is enough? Why or why not?
   (ii) What are the areas that you think need further improvement? What suggestions would you make to the government for assisting parents with autistic children? Why?

4. (i) Do you think the support from the schools is adequate? Why or why not?
   (ii) What kind of support could schools provide to parents with autistic children?
